# Supplementary material for: Chromosome-scale genome assembly of acerola (Malpighia emarginata DC.)
Source: DNA Res. 2024 Oct 7;31(6):dsae029. doi: 10.1093/dnares/dsae029 (PMC11555059; doi:10.1093/dnares/dsae029)
Supplement: dsae029_suppl_Supplementary_Figures_S1-S3 [file dsae029_suppl_supplementary_figures_s1-s3.pdf]

Supplementary Information

Chromosome-scale genome assembly of acerola (*Malpighia emarginata* DC.)

Kenta Shirasawa[1*], Kazuhiko Harada[2], Noriaki Haramoto[2], Hitoshi Aoki[2], Shota Kammera[3], Masashi Yamamoto[3], Yu Nishizawa[3]

[1]Kazusa DNA Research Institute, Chiba 292-0818, Japan

[2]Nichirei Foods Inc., Chiba 261-0002, Japan

[3]Faculty of Agriculture, Kagoshima University, Kagoshima 890-0065, Japan

*To whom correspondence should be addressed:

Kenta Shirasawa

Tel.: +81-438-52-3935

Fax.: +81-438-52-3934

Email: shirasaw@kazusa.or.jp

Supplementary Table S1 Number of ddRAD-Seq reads, map rate on the reference sequence, and accession numbers of ddRAD-Seq for an S1 mapping population via self-pollination of NRA309.

Supplementary Table S2 Number of ddRAD-Seq reads, map rate on the reference sequence, and accession numbers of breeding materials.

Supplementary Table S3 Reference genomes used for removing organelle sequences from the acerola genome assembly.

Supplementary Table S4 Functional annotation for the predicted genes in the acerola genome.

Supplementary Table S5 Annotation of variants detected among 60 acerola lines.

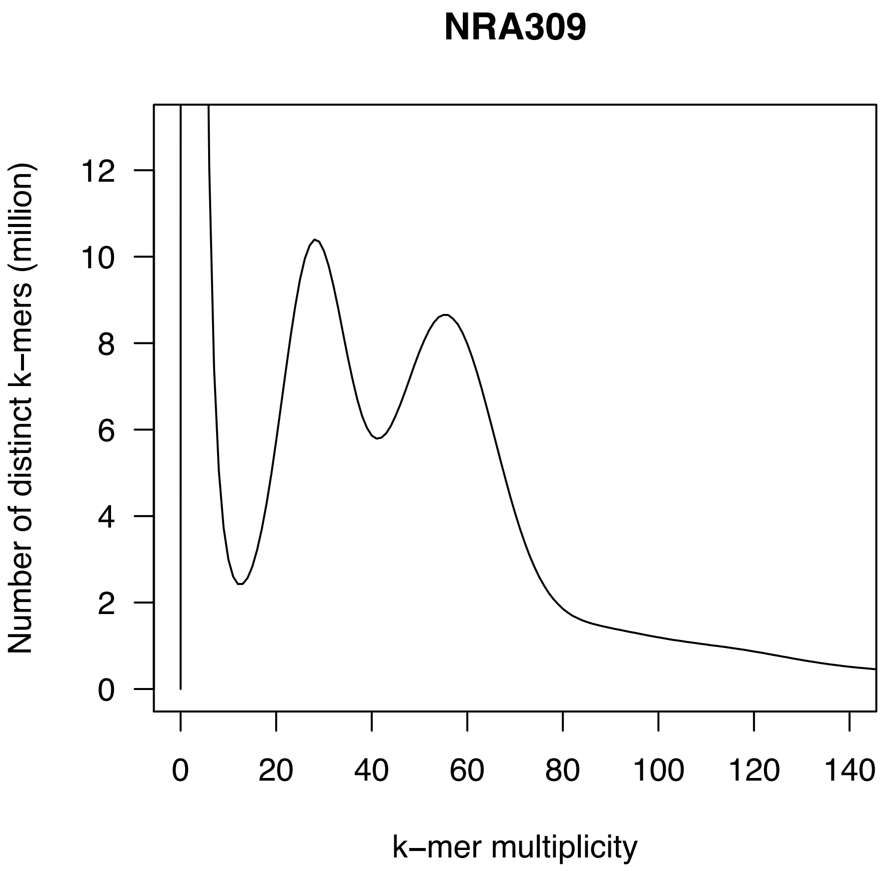

Supplementary Figure S1 Estimated size of the acerola genome, based on *k*-mer analysis (*k* = 17), with the given multiplicity values.

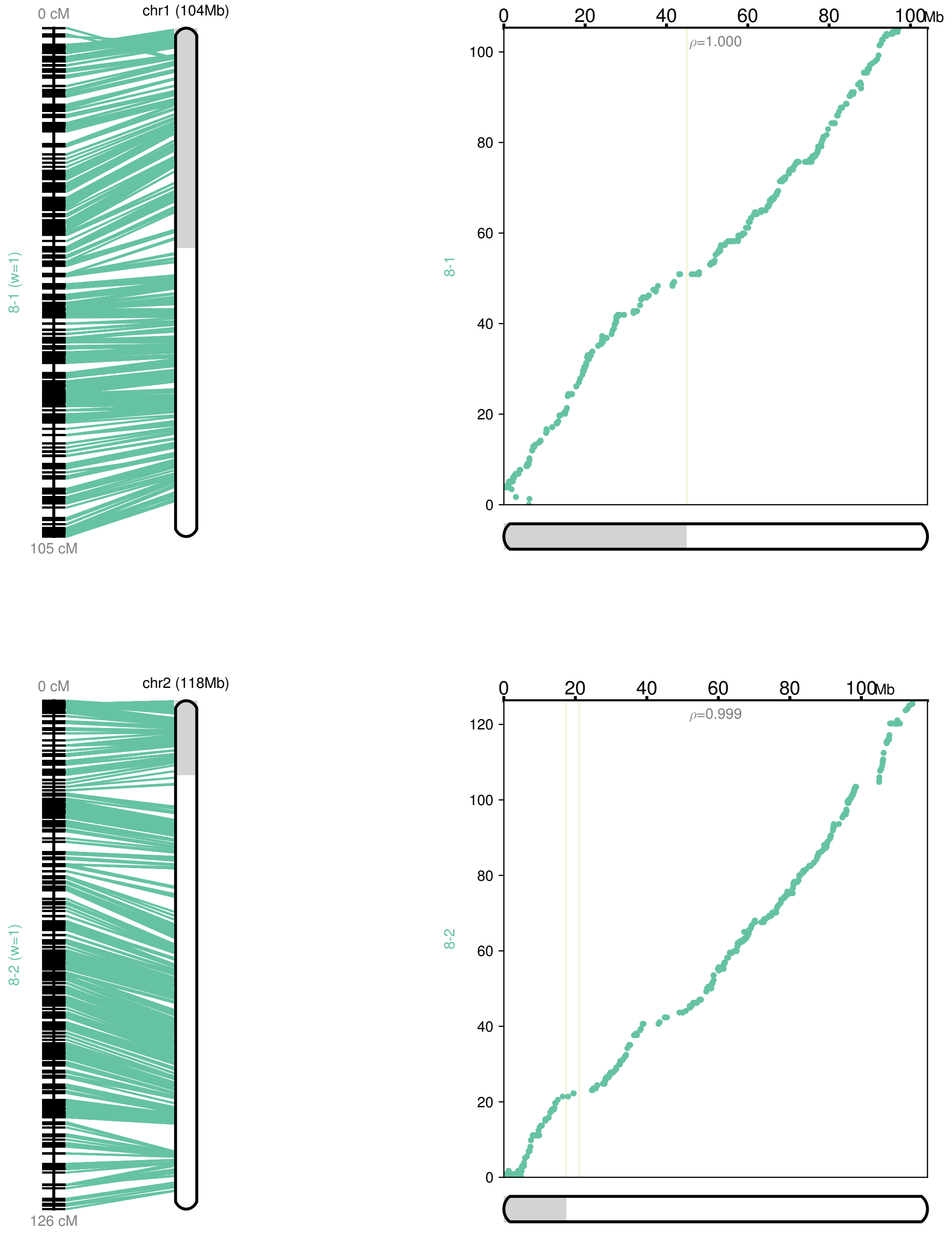

Supplementary Figure S2 Genetic and physical maps of the acerola genome.

Left: SNP loci on the genetic map (vertical lines) and physical map (bars) are connected with horizontal lines. Right: Positions of SNP loci are indicated with dots on the genetic map (y-axis, cM) and physical map (x-axis, Mb). The vertical yellow lines indicate boarders of contigs.

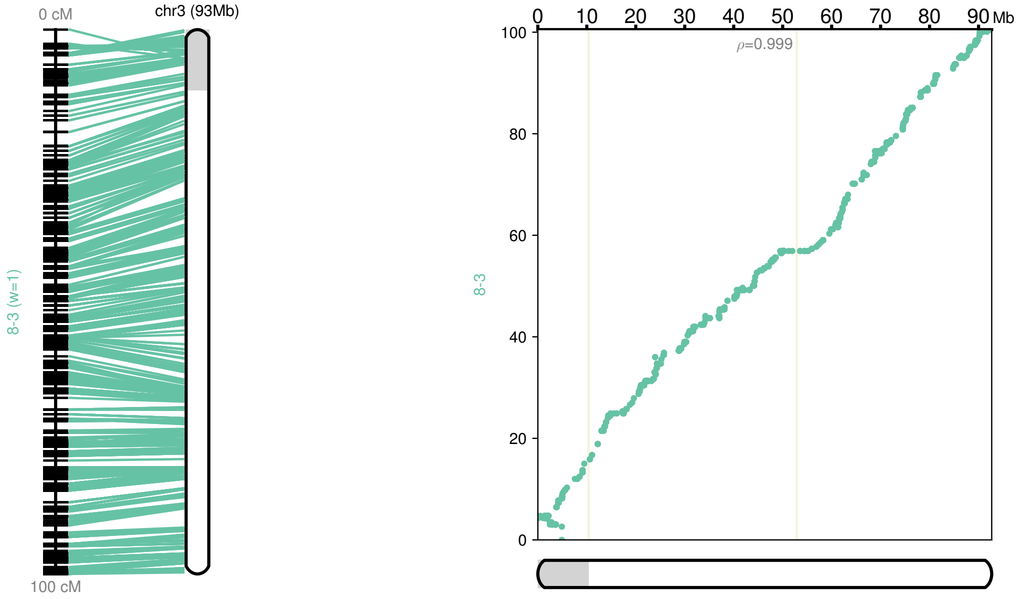

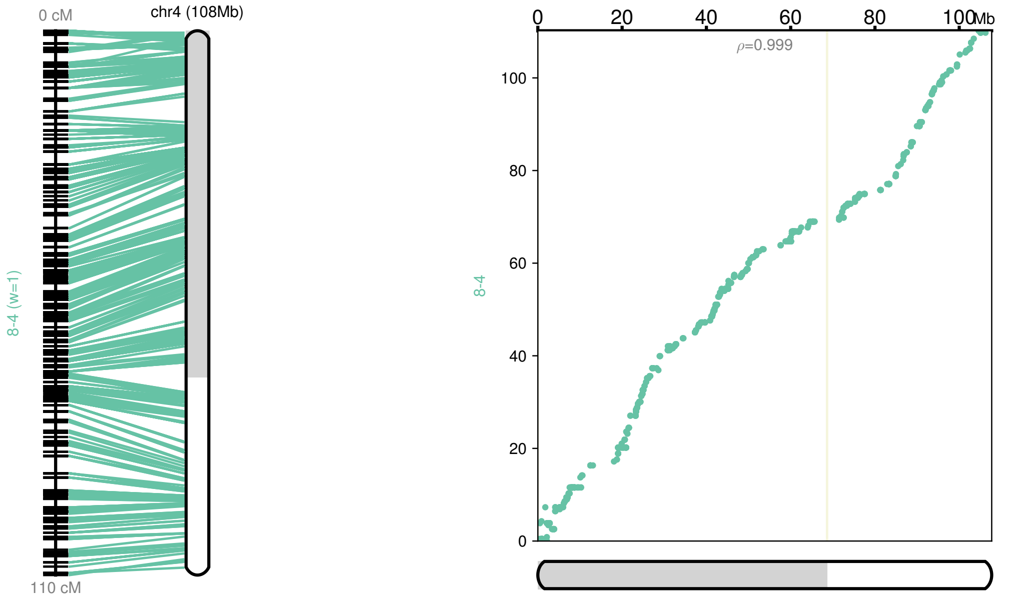

Supplementary Figure S2 (continued)

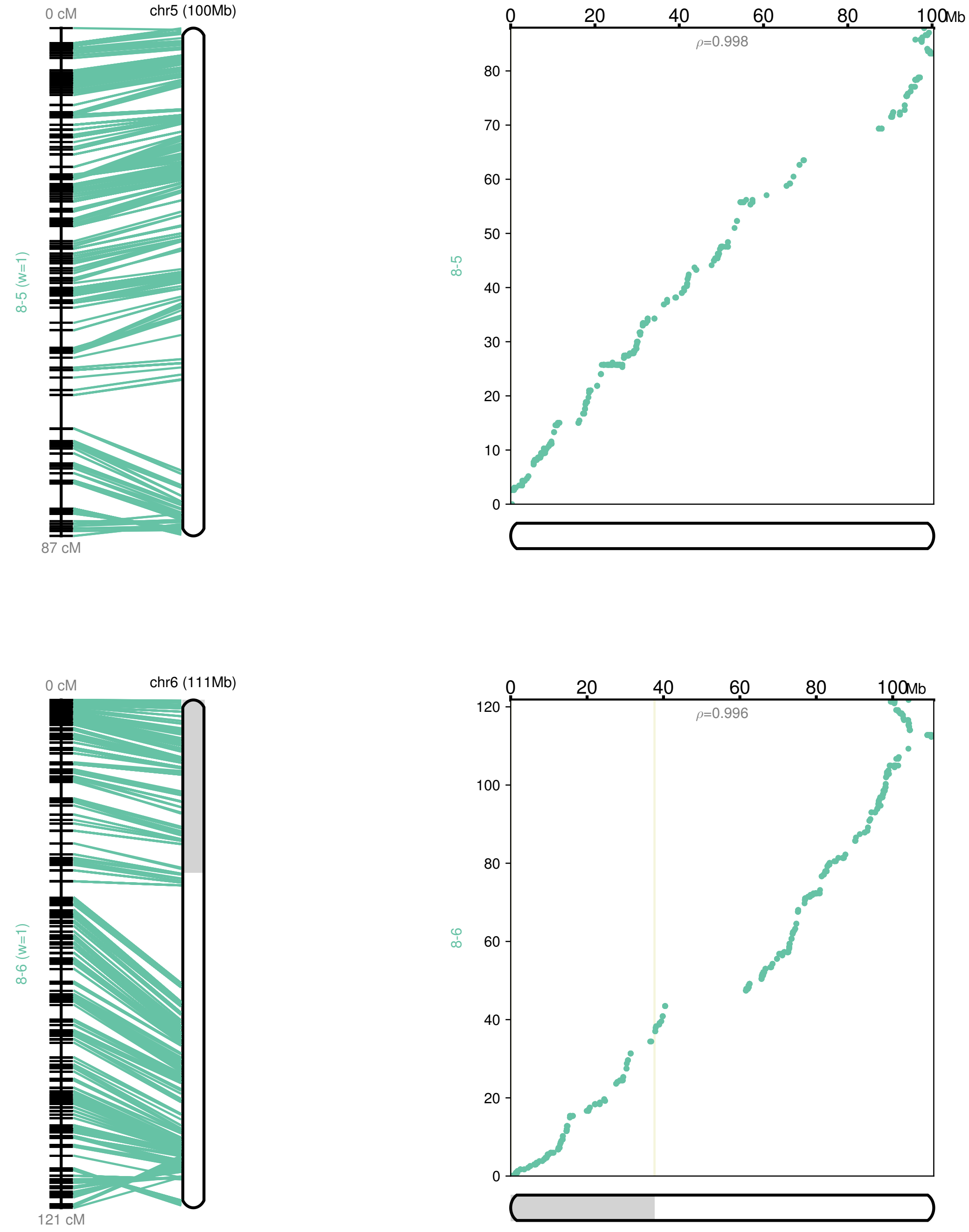

Supplementary Figure S2 (continued)

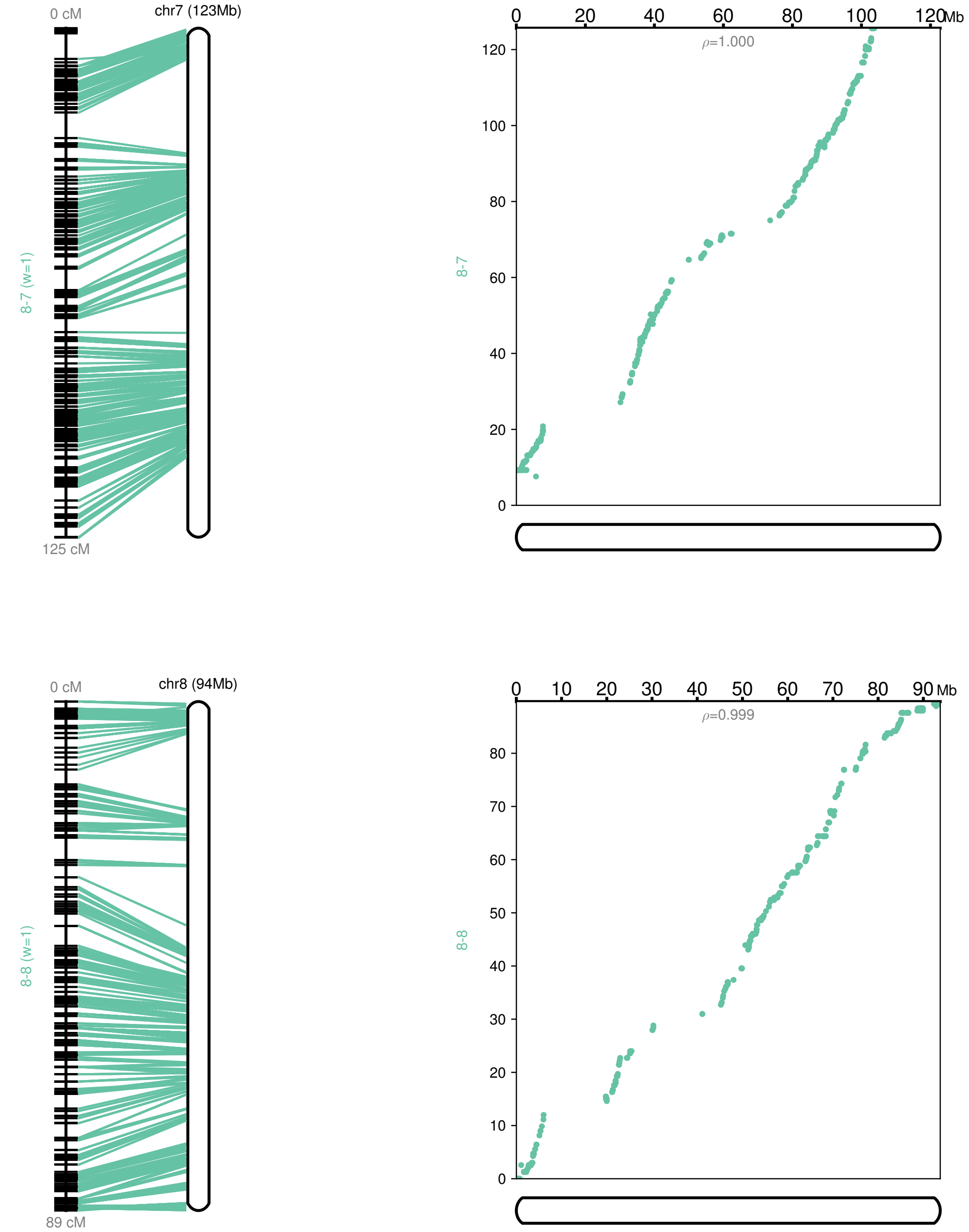

Supplementary Figure S2 (continued)

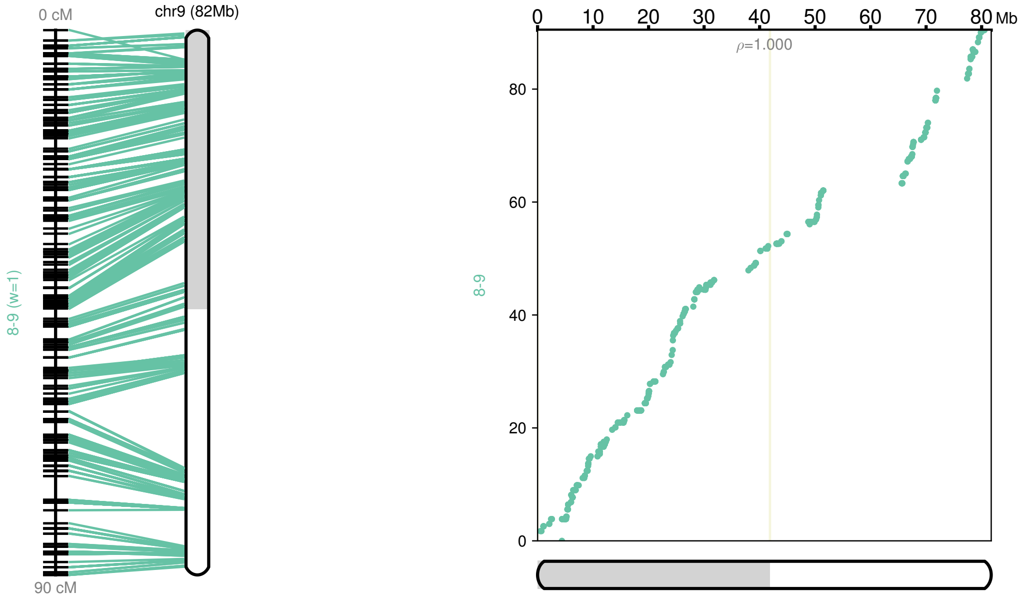

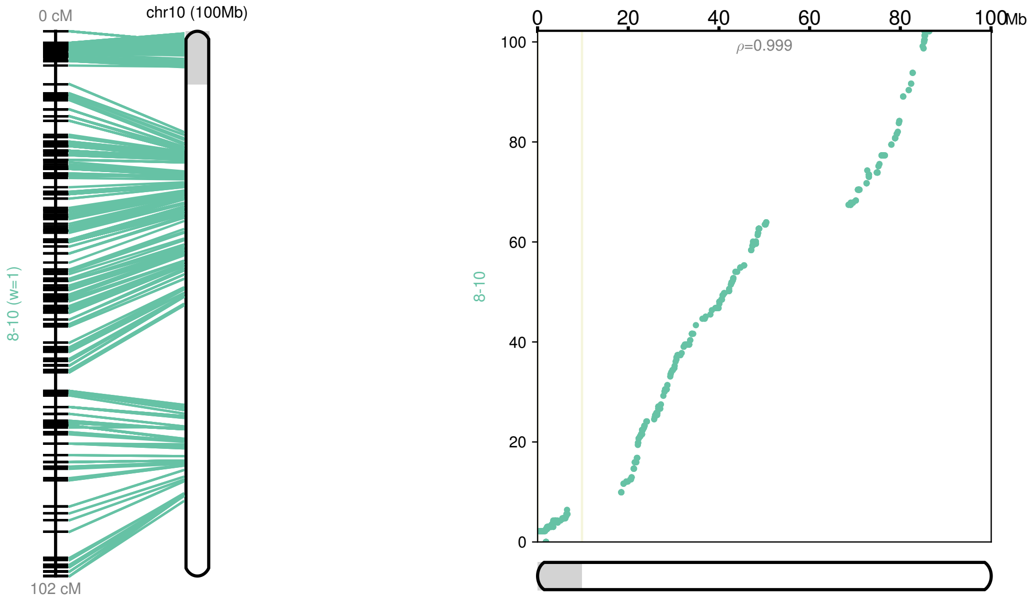

Supplementary Figure S2 (continued)

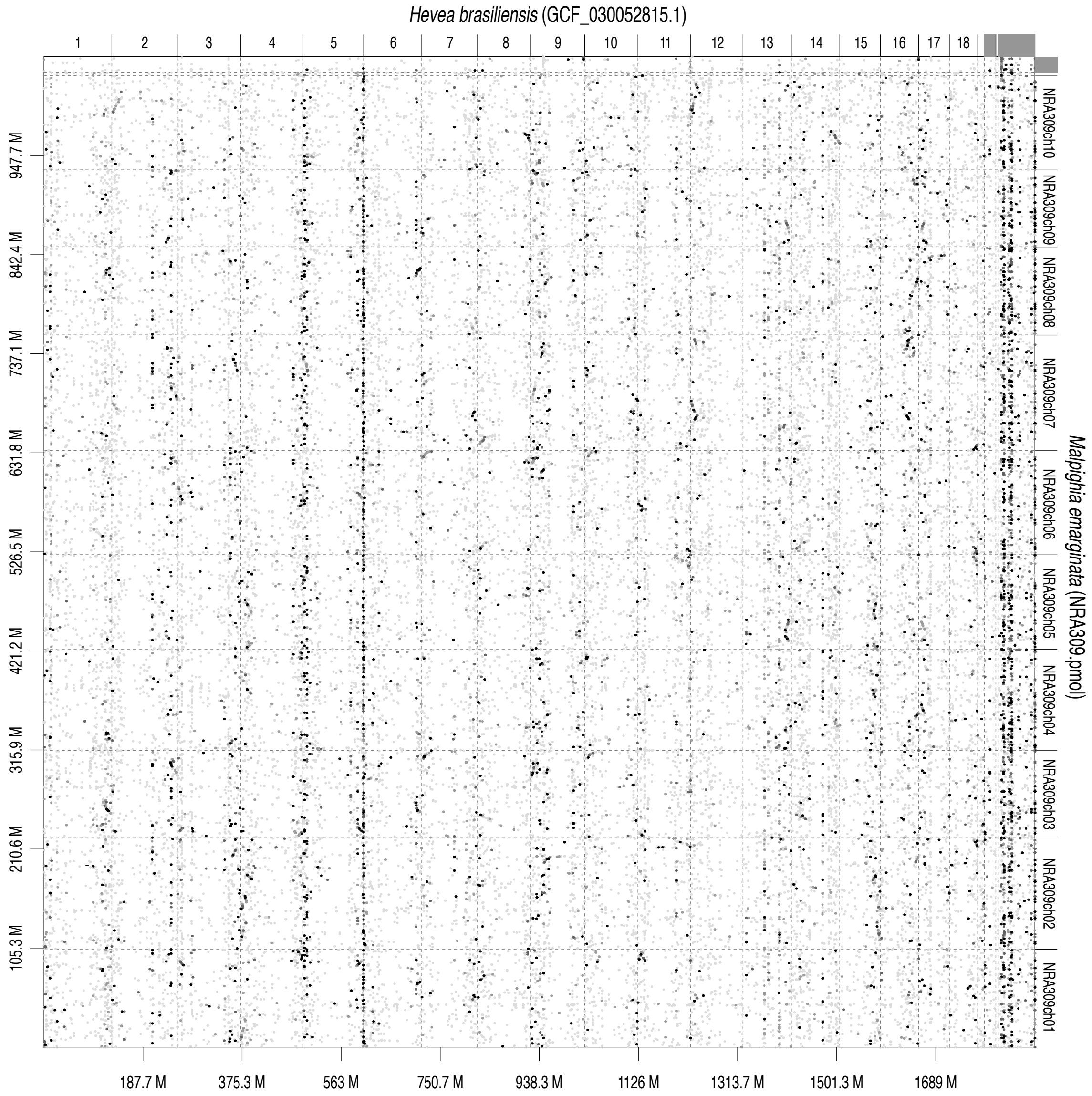

Supplementary Figure S3 Comparative analysis of the genome sequence and structure of acerola and three other Malpighiales species.

Dots indicate similarities in the genome structures of rubber tree (*Hevea brasiliensis*), cassava (*Manihot esculenta*), and castor bean (*Ricinus communis*). Chromosome numbers are indicated above the x-axis and on the right side of the y-axis, and genome sizes (Mb) are shown below the x-axis and on the left side of the y-axis.

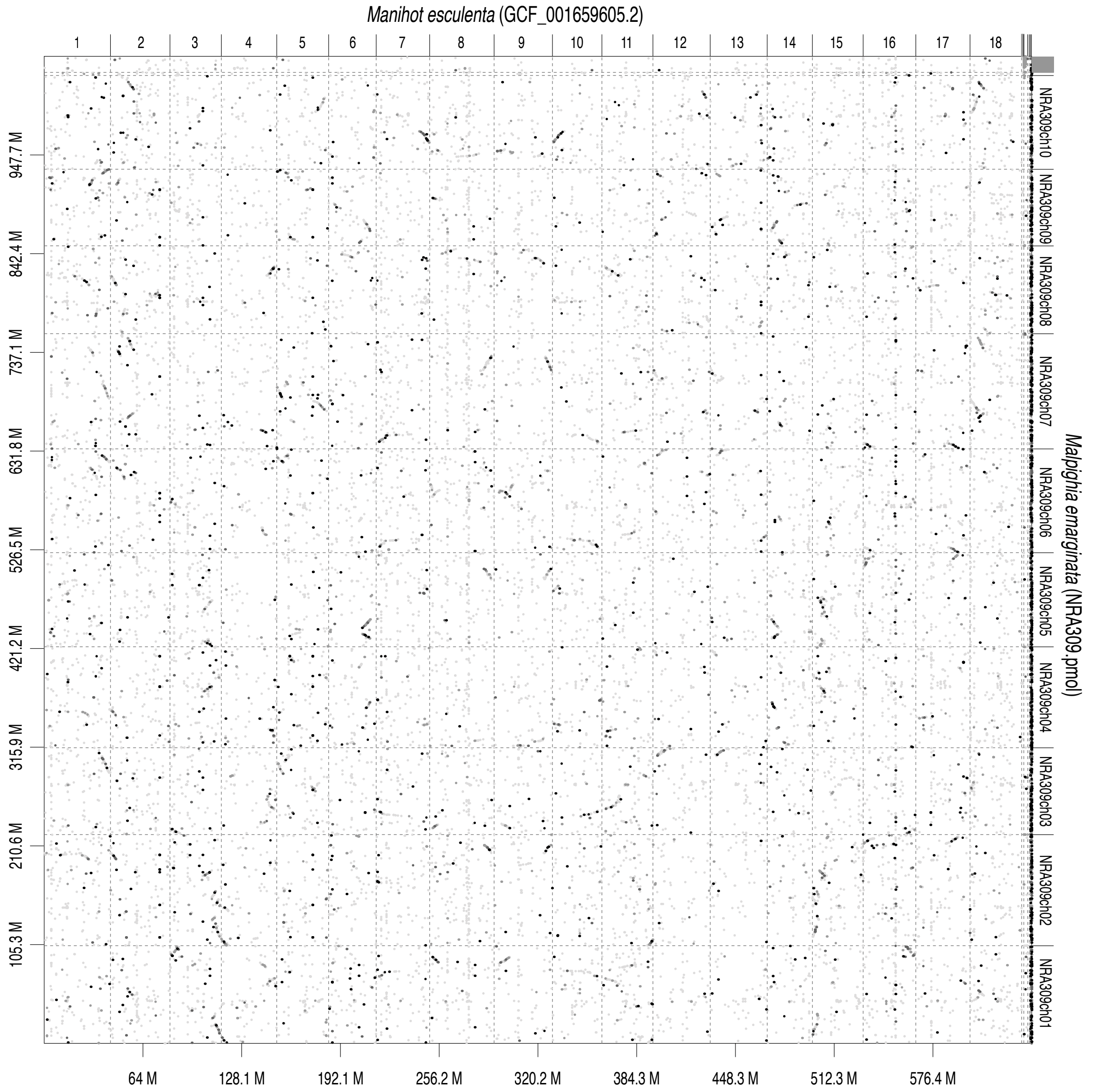

Supplementary Figure S3 (continued)

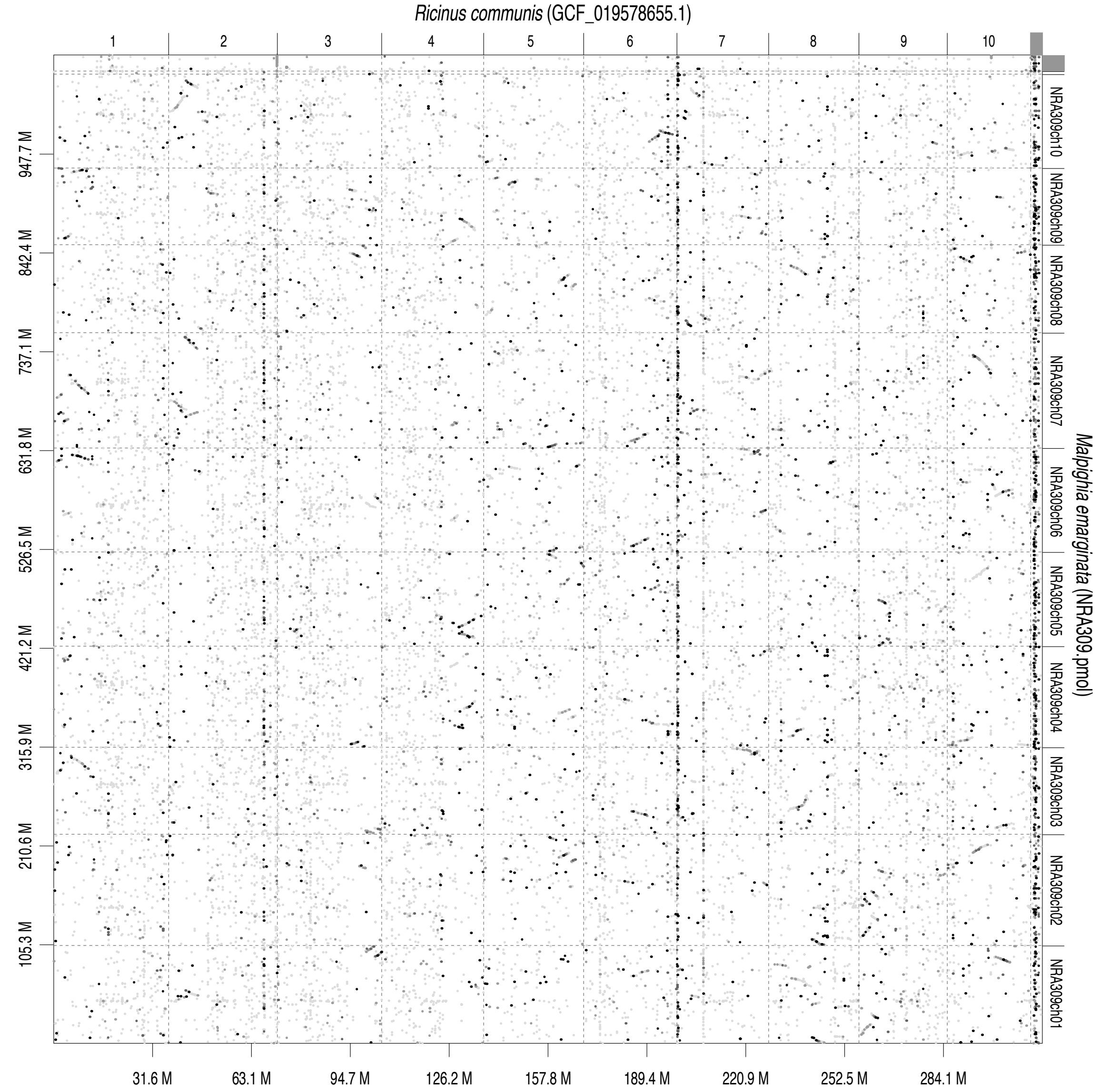

Supplementary Figure S3 (continued)
